# Behavioural activation for low mood and anxiety in male frontline NHS workers (BALM): a pre-post intervention study

**DOI:** 10.1136/bmjopen-2024-094214

**Published:** 2025-06-12

**Authors:** Paul Galdas, Della Bailey, Steve Bell, Katharine Bosanquet, Carolyn Chew-Graham, David Ekers, Simon Gilbody, Elizabeth Littlewood, Michael Mawhinney, Heidi Stevens, Katie Webb, Dean McMillan

**Affiliations:** 1 Department of Health Sciences, University of York, York, England, UK; 2 Medical Directorate, North West Ambulance Service NHS Trust, Bolton, UK; 3 University of Keele, Keele, UK; 4 Psychiatry, University of York, York, UK; 5 Northern Ireland Hospice, Newtownabbey, Antrim, UK

**Keywords:** MENTAL HEALTH, Health Workforce, Psychosocial Intervention

## Abstract

**Objectives:**

To evaluate the impact and acceptability of a tailored, gender-responsive behavioural activation (BA) intervention for improving depression and anxiety in male National Health Service (NHS) frontline workers.

**Design:**

Pre-post intervention study.

**Setting:**

Three NHS organisations in the North of England.

**Participants:**

45 men aged ≥18 years working in a frontline NHS role scoring in the subclinical range (5–14) on the Patient Health Questionnaire-9 (PHQ-9) (depression) and/or the Generalised Anxiety Disorder-7 (GAD-7) (anxiety) at baseline.

**Interventions:**

A tailored BA treatment programme consisting of up to eight telephone support sessions over a period of 4–6 weeks, accompanied by a BA self-help manual.

**Main outcome measures:**

Self-reported symptom severity of depression, assessed by PHQ-9, and anxiety, assessed by GAD-7, at baseline and 4 and 6 months. Acceptability from the perspectives of male study participants and coaches who delivered the intervention was assessed in a nested qualitative study using the theoretical framework of acceptability (TFA).

**Results:**

PHQ-9 and GAD-7 scores decreased from baseline to 4 months on both the PHQ-9 and GAD-7. While scores increased from 4 months to 6 months, the 6-month scores remained below those of the baseline scores. Acceptability of the intervention was high across all constructs of the TFA. The practical and action-oriented strategies of the intervention, and the confidential, flexible, convenient mode of delivery, worked to support men’s engagement with the intervention.

**Conclusions:**

Delivery of a tailored, gender-responsive BA intervention was appealing to, and beneficial for, men working in frontline NHS roles with less severe depression and anxiety. The BALM intervention offers promise as a tailored workplace mental health programme that is aligned with men’s needs and preferences and can help overcome a reticence to engage with mental health support in NHS staff and beyond.

**Trial registration number:**

ISRCTN48636092.

**STRENGTHS AND LIMITATIONS OF THIS STUDY**

- The study used a single-sample, pre-post design with embedded qualitative methods to explore acceptability.
- Data collection and analysis were informed by the theoretical framework of acceptability, enabling a systematic and theory-driven evaluation.
- Acceptability data included interviews with men who did not complete the intervention, providing insight into potential barriers to engagement.
- Participants were a self-selecting sample who may have been particularly motivated to take part, and the lack of ethnocultural diversity and representation from some NHS staff groups, particularly ancillary roles, limits the transferability of findings.
- The quasi-experimental research design is not sufficiently reliable to make causal inferences.

## Introduction

 There are unprecedented and sustained high levels of sickness absence in the National Health Service (NHS) workforce in England, with increases observed across all staff groups since 2009. Rates rose sharply during the coronavirus disease 2019 (COVID-19) pandemic and have remained elevated compared with pre-pandemic levels.^1^

The major cause of sickness absence in NHS staff is mental health conditions, particularly anxiety and depression, which now account for around a quarter of all sick days.^1 2^ Approximately 6 million sick days for mental health and well-being-related reasons were reported in 2022, an increase of 26% since 2019.^2^ By contrast, mental illness accounts for 8%–12% of sickness absence each year in the broader UK labour force, meaning NHS employees are two to three times more likely to require sickness absence leave for mental ill health than the average UK employee.^1^ Sickness absence of healthcare professionals can lead to suboptimal patient care and significant increased costs and is increasingly a cause for staff to leave the NHS.^1 3^ As a result, there is growing interest in interventions to support the mental health and well-being of healthcare workers, globally.^3^

Frontline healthcare staff are exposed to a variety of workplace stressors throughout their careers that can lead to mental distress.^4^ Causes vary, but high workload, long hours, difficult shift patterns and the absence of healthy rotation schedules that accommodate adequate rest, sleep and restoration over time have been consistently identified in the empirical literature as important contributors to much of the common mental health challenges faced by healthcare professionals.^4 5^ This distress and these adverse psychological outcomes do not typically require high-intensity mental health interventions.^6^ A number of ‘low-intensity’ strategies have been recommended; these include supporting regular breaks, adequate rest and sleep, a healthy diet, physical activity, peer and family support, avoidance of unhelpful coping strategies (eg, use of alcohol and drugs), limitation of social media use, mindfulness and meditation practices, and professional counselling or psychological services.^3 5^ However, uncertainty exists about the best models of care to effect positive change in this population. Thus far, workplace interventions have failed to produce a continuous downward trend in sickness absence rates.^7^ Barriers to uptake and engagement have also been reported, in particular frontline workers, or the organisations in which they work, are not being fully aware of what they need to support their mental well-being.^5^

Relevant here is the culture in many frontline healthcare and emergency professions that has promoted the idea that they are (or should be) stronger and more resilient than the general population.^8^ This ethos can impede the willingness of staff to acknowledge psychological problems and seek support, and may be particularly problematic for male frontline workers by adding to the stigma of help seeking for mental health concerns found in the general population.^8 9^ Traditional masculine norms characterised by self-reliance, stoicism and restrictive emotionality are associated with higher stigmatisation of mental health problems and reluctance to acknowledge or seek help from professional mental health services.^10^ Although the tailoring of psychological support to address these issues has been a long-standing topic of concern in the men’s health literature, little evidence exists on effective gender-responsive interventions (ie, those which pay attention to men’s specific needs, gender-based barriers and gender differences) in the general workforce or frontline healthcare communities.^8 11^ To address this gap in the evidence, the current study aimed to develop, deliver and evaluate a gender-responsive mental health intervention for low mood and anxiety in male frontline NHS workers, based on behavioural activation (BA) principles.

BA is a brief, evidence-based, guided self-help treatment that offers promise as a gender-responsive approach to early mental health intervention for men due to its practical, collaborative and action-oriented strategies that are consistent with a strengths-based masculinities approach that reinforces participants’ sense of autonomy, control and independence.^11 12^ The evidence for the use of BA as an early intervention in people without a formal mental health diagnosis is strong. National Institute for Health and Care Excellence (NICE) guidance in the UK^13^ recommends BA as a first-line treatment option for adults with less severe depressive symptoms (ie, mild depression and those with ‘subthreshold’ symptoms which fall below the criteria for diagnosis) and significant effects have also been reported for improving quality of life and anxiety.^14^ However, to our knowledge, no study has evaluated the impact and acceptability of BA as a workplace mental health intervention for men in either the general population or in frontline healthcare staff.

The specific objectives of this study were to (1) evaluate the impact of a tailored, gender-responsive BA intervention for improving depression and anxiety in male NHS frontline workers and (2) explore the acceptability of the intervention from the perspective of male study participants and the coaches who delivered it.

## Methods

### Study design

We used a single-sample, pre-post study design to assess baseline depression and anxiety measures in male frontline NHS workers and determine the impact of the intervention on their mental health at 4 and 6 months, with a nested qualitative study using semistructured interviews to explore the acceptability of the intervention from the perspectives of male study participants and the coaches who delivered it. The 4-month and 6-month follow-up time points were selected to assess both short-term and medium-term changes in mental health outcomes, consistent with similar studies of brief psychological interventions in workplace and primary care settings.^15^ While the 6-month follow-up provided some insight into the maintenance of effects, longer-term outcomes were beyond the scope of this study.

### Participants and sample

Participants were recruited from three NHS organisations (‘Trusts’) in the North of England: two hospital Trusts and an ambulance service. To be eligible for the study, participants had to identify as male, be aged ≥18 years and work in a ‘frontline’ NHS role as defined in NHS Workforce Statistics.^16^ Eligible staff groups included professionally qualified clinical staff, support to clinical staff and NHS infrastructure support staff.

Participation was voluntary and selected as a convenience cluster sample. Study information was distributed via posters displayed in staff areas and electronic bulletins/staff intranet across the three study sites, and through social media platforms. Potential participants were invited to visit a secure online site to complete a consent form if they were interested in taking part after reading study materials, followed by a baseline questionnaire assessing their eligibility. As BALM is an early mental health intervention, individuals were eligible for participation in this study if they scored in the subclinical range (5–14) on the Patient Health Questionnaire-9 (PHQ-9)^17^ and/or the Generalised Anxiety Disorder-7 questionnaire (GAD-7)^18^ at baseline. Individuals scoring outside this range, currently receiving treatment for a mental health condition, or who had been previously diagnosed with bipolar disorder, schizophrenia or other psychotic disorder, were excluded.

Following completion of the baseline questionnaire, eligible participants were sent a letter via email to confirm their participation. Those who did not meet the eligibility criteria were sent a letter thanking them for their interest in the study, including signposting to other mental health and well-being support programmes, or referred to their GP, as appropriate.

### Intervention

The intervention, ‘BALM’, is a structured programme of BA tailored to specifically meet the needs of male NHS workers with less severe depression and/or anxiety symptoms, through the application of a gendered lens.^19^ BA is a practical treatment that explores how life events can result in a reduction of meaningful activity, which in turn leads to feelings of low mood and anxiety. In response to these feelings, a person may attempt to cope through avoidance. While this may work in the short term, in the long term, it may lead to further reductions in important or meaningful activity. In the current study, life events were principally related to both workplace stressors, such as high workload, long hours and difficult shift patterns, but could also include non-workplace stressors such as changes in routine or life circumstances, relationships and financial pressures. The intervention consisted of a coach and participant working together to develop a collaborative treatment plan that sought to increase meaningful activities and reduce avoidance.

The process of intervention tailoring for BALM involved a stakeholder advisory group comprising NHS psychological well-being practitioners and male NHS workers from medicine, paramedicine, nursing, allied health and ancillary occupational groups. The group met on three occasions in co-design workshops to tailor aspects of the intervention relating to context, content and communication in accordance with concepts outlined in the 5C Framework for designing men’s health programmes.^19^ The main adaptations related to the format, design, content and use of language in the self-help manual, and the content of the coach training to incorporate evidence-based guidance on ways to engage men in mental health support.

The final BALM intervention consisted of up to eight telephone or video call support sessions over a period of 4–6 weeks, accompanied by a structured self-help manual grounded in BA principles and tailored for men using gender-responsive strategies.^19^ The manual, developed through co-design workshops with NHS staff and mental health practitioners, is organised into five stages that reflect key elements of the intervention. These stages formed the basis for the support sessions, with progress through the manual guided by individual needs and preferences (see table 1).

**Table 1:** Overview of the core BALM intervention content

Stage	Focus	Key components
Stage 1: the importance of behaviour	Understanding the link between behaviour and mood	Recognising symptoms; introduction to the behavioural activation cycle
Stage 2: making a note of what you do	Monitoring current activity and its impact	Activity diary; exploring how behaviours affect mood
Stage 3: setting goals	Establishing direction and motivation	Identifying personal goals for the intervention period
Stage 4: follow the plan, not the mood	Initiating behavioural change	Activity planning; addressing avoidance; problem-solving; worry postponement
Stage 5: keeping going with the changes	Planning for long-term maintenance	Coping with setbacks; early warning signs; relapse prevention

Support sessions lasted approximately 30 min and were facilitated by coaches voluntarily recruited from the three NHS Trust study sites. Both male and female coaches were recruited from a broad range of clinical and non-clinical NHS backgrounds, including psychological well-being practitioners, nurses, paramedics, research assistants, clinical support officers and crisis support workers. Coaches were trained to deliver the intervention and review progress and outcomes in three online full-day workshops. In response to feedback from the co-design workshops, BALM participants were allocated a coach from a different NHS employer to their own to help to overcome barriers to engagement by maintaining confidentiality and anonymity. A secure computer system was used to monitor care, and supervision of coaches was provided by DM and DB. While participants could engage in up to eight support sessions, the pace, structure and focus of each session were flexible. The number of sessions, total duration of the intervention, and mode of delivery (telephone or video call) were not standardised across the sample and varied between participants according to individual needs and preferences. Data on session duration, number of sessions, and differences in outcomes by delivery mode were not collected.

### Outcomes

We studied outcomes of self-reported symptom severity of depression, assessed by PHQ-9,^17^ and anxiety, assessed by GAD-7.^18^ The PHQ-9 is a 9-item instrument that assesses the frequency of depressive symptoms over the past 2 weeks, with scores ranging from 0 to 27; higher scores indicate greater symptom severity.^17^ The GAD-7 is a 7-item instrument measuring generalised anxiety symptoms over the past 2 weeks, with scores ranging from 0 to 21.^18^ Both instruments are widely used in clinical and research settings and have demonstrated strong reliability and validity. Health-related quality of life was assessed using the 12-item Short Form Health Survey (V.2) (SF-12 V.2), which generates physical and mental health composite scores. Scores are standardised to a mean of 50 and a SD of 10 in the general population, with higher scores indicating better health status.^20^ Acceptability from the perspectives of male study participants and coaches who delivered the intervention was assessed in a nested qualitative study based on the seven component constructs of the theoretical framework of acceptability (TFA).^21^ Additional pre-specified secondary outcomes, consistent with the trial registry (ISRCTN48636092), included levels of uptake, adherence and drop-out. These were assessed using recruitment and retention data (eg, enrolment, withdrawal and completion rates), and explored further through qualitative interviews with both completers and non-completers.

### Sample size

As BA is well established as an effective intervention for less severe depression,^14 22^ overall sample size was determined by pragmatic considerations with the aims of (1) benchmarking the pre-post results against the pre-post effects seen in the intervention and control arms of trials of BA delivered using a similar format and (2) achieving meaning saturation (ie, a comprehensive understanding of the issues raised in the data^23^) in the qualitative analysis of acceptability. We specified an enrolment target of 45 individuals as we believed this would achieve these aims.

### Data collection

Study participants were invited to complete the PHQ-9 and GAD-7 questionnaires online via Qualtrics^24^ at baseline (pre-treatment) and after 4 and 6 months. At baseline, participants were also asked to provide demographic information which included age, ethnicity, employer, occupation and number of years worked in the NHS.

Qualitative data were collected by KW, HS and KB in one-to-one semistructured interviews conducted via telephone or video call (according to individual preference) with participants and coaches. None of the interviewers had acted as coaches for the participants they interviewed. Interviews explored experiences of the intervention and thoughts around content and delivery, including barriers and facilitators to following the treatment plan. Questions followed a topic guide developed by the study team informed by the TFA and input from the stakeholder advisory group, who provided guidance on the content and phrasing of questions. All interviews were recorded using an encrypted digital voice recorder, and participants were assigned an anonymous identifier. Recordings were transcribed verbatim using a professional transcription service and uploaded to NVIVO V.12.^25^ In total, 32 study participants were selected via consecutive sampling and interviewed from the pool of 45 study participants who consented to take part in an interview at initial recruitment: 24 were ‘completers’ and 8 were ‘non-completers’. 12 coaches were also selected for interview.

### Analysis

#### Quantitative analysis

Pre-post effect sizes and associated 95% CI were calculated for the PHQ-9 and GAD-7. A standardised mean difference, Hedges’ g (repeated measures), is reported rather than a mean difference to facilitate comparison of the results with other studies. Separate effect sizes were calculated for baseline to 4 months and baseline to 6 months. Analyses were conducted using a completer approach, based on participants who provided data at each time point. For an effect size to be calculated, data were needed for each time point on the measure. In the absence of a control condition, data were benchmarked against the CASPER trial.^15^ Although the CASPER trial has a different population (older adults) and setting (recruitment from primary care), it is comparable to the BALM study in a number of ways: it too uses a brief, guided self-help intervention, the intervention is based on BA principles, the samples had subclinical levels of symptomatology, and the same follow-up point was used (4 months).

#### Qualitative analysis

**Figure 1:**
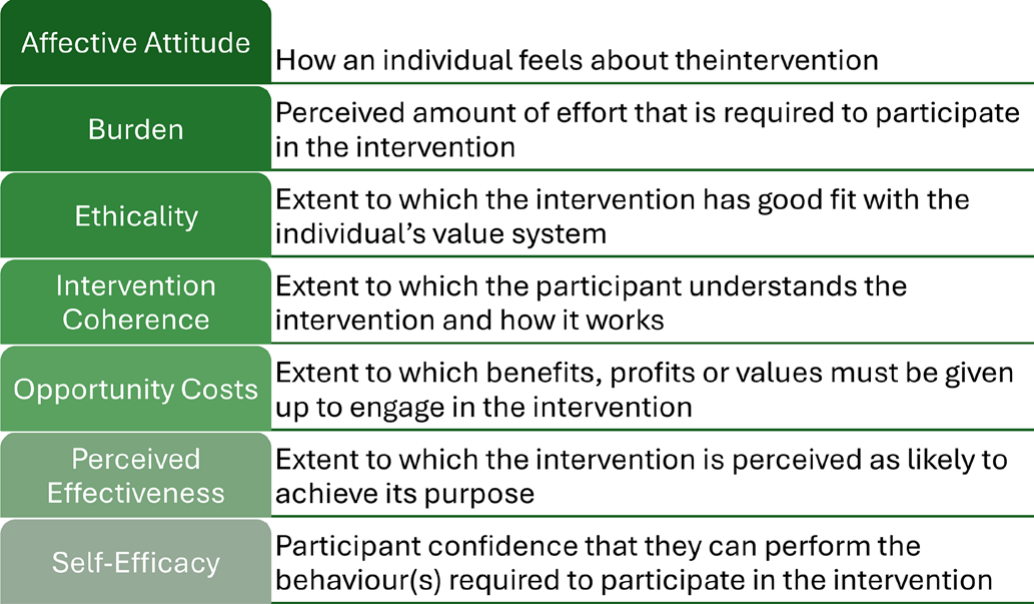
The seven component constructs of the theoretical framework of acceptability (TFA).^21^

Qualitative data were analysed thematically using a Framework Approach.^26^ An a priori coding frame was developed based on acceptability/satisfaction with the intervention as defined by the TFA^21^ (figure 1), while also allowing for the emergence and exploration of any unanticipated themes. Initial analysis involved coding within each transcript followed by analysis across the codes and transcripts. Initial coding and analysis were conducted independently by KB, HS, PG and KW, with team discussion to agree final analysis and identify discrepancies. Participant and coach data were analysed separately following the same analytical process.

## Results

### Recruitment and participant flow

**Figure 2:**
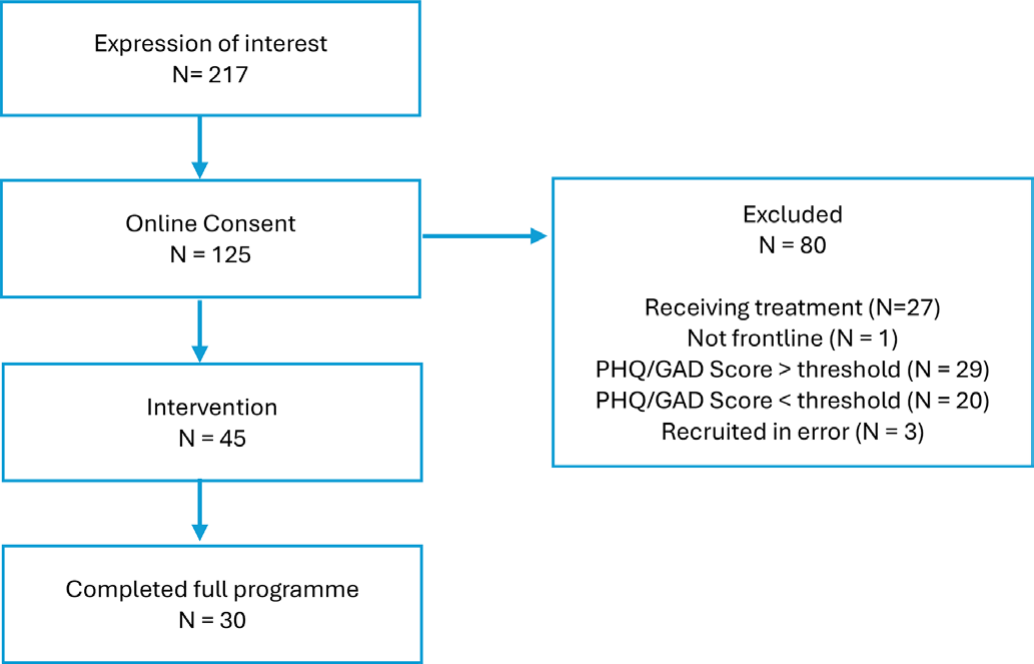
Participant flow diagram. GAD, Generalised Anxiety Disorder; PHQ, Patient Health Questionnaire.

Recruitment took place in the period January to September 2023. 217 potential participants expressed interest in taking part in the study, of whom 45 were enrolled (see figure 2). Recruitment ceased when the enrolment target of 45 individuals was reached. Of these, 30 completed the full BALM programme (‘completers’) and 15 did not complete the full intervention (‘non-completers’). Completer status was defined as attending at least four coach sessions, which was typically the minimum required for participants to progress through the five structured stages of the BALM intervention. This resulted in an intervention completion rate of 67%, which is comparable to larger scale trials of BA in heterogeneous clinical populations.^14^ These figures reflect the pre-specified secondary outcomes of uptake, adherence and drop-out, as outlined in the trial registry.

### Baseline data

Demographic and clinical characteristics (PHQ-9 and GAD-7) were gathered at baseline from male study participants. The mean age of the study participants was 40.8 years (SD=11.0). All men self-identified as white/Caucasian. The majority of the men (61.9%) were married or in a domestic partnership. Male participants came from a range of NHS roles: nursing (n=11), ambulance service team (n=8), allied health professionals (n=7), psychological professions (n=6), management (n=4), doctors (n=3), healthcare support workers (n=3) and other (n=3). The mean number of years working in the healthcare sector was 10.5 (SD=9.2).

The mean age of the coaches (n=16) was 49.2 years (SD=10.5). The majority were female (n=10). All coaches self-identified as white/Caucasian. Coaches came from a range of NHS roles: allied health professionals (n=6), admin and estates staff (n=4), ambulance service team (n=3), nursing (n=1), management (n=1) and healthcare science (n=1).

### Quantitative outcomes

Table 2 summarises male study participant scores on the PHQ-9, GAD-7 and SF-12 at baseline, 4 months and 6 months. The higher number of participants providing outcome data at 6 months reflects the flexible, rolling nature of data collection, with some participants completing the 6-month follow-up despite missing the 4-month assessment. Scores decreased from baseline to 4 months on both the PHQ-9 and GAD-7. While scores increased from 4 months to 6 months on the PHQ-9 and GAD-7, the 6-month scores remained below those of the baseline scores. The SF-12 physical component score reduced (meaning worse health) at 4 months then returned to a score similar to baseline at 6 months. The SF-12 mental component score increased (meaning better health) at 4 months, and while it decreased between 4 and 6 months, it remained above the baseline score.

**Table 2:** PHQ-9 and GAD-7 scores at baseline, 4 months and 6 months

Timepoint	PHQ-9Mean (SD)	GAD-7Mean (SD)	SF-12 (physical component)mean (SD)	SF-12 (mental component)mean (SD)
Baseline (n=36)	7.5 (2.82)	6.0 (2.64)	54.5 (6.6)	35.7 (9.2)
Four months (n=30)	4.17 (4.18)	2.97 (2.97)	51.6 (7.4)	46.3 (9.0)
Six months (n=37)	5.11 (4.82)	4.11 (4.23)	54.8 (6.0)	42.9 (11.6)

GAD-7, Generalized Anxiety Disorder-7; PHQ-9, Patient Health Questionnaire-9; SF-12, 12-item Short-Form Health Survey (version 2).

The pre-post effect size (repeated measures Hedges’ g) at 4 months on the PHQ-9 was 0.86 (95% CI=0.46–1.30) and at 6 months was 0.88 (95% CI=0.51–1.28). For the GAD-7, the 4-month effect size was 1.0 (95% CI=0.52–1.50) and at 6 months was 0.63 (95% CI 0.19 to 1.08). For the SF-12 physical component score, the 4-month effect size was 0.40 (95% CI 0.03 to 0.79) and effect size at 6 months was −0.07 (95% CI −0.38 to 0.23). For the SF-12 mental component score, the 4-month effect size was −1.13 (95% CI −1.70 to −0.62) and effect size at 6 months was −0.67 (95% CI −1.03 to −0.34). The 4-month figures were benchmarked against the 4-month pre-post effect sizes for the intervention and control conditions in the CASPER trial (see table 3).

**Table 3:** Benchmarking BALM pre-post effect sizes at 4 months with CASPER trial

	BALMHedges’ g (95% CI)	CASPER interventionHedges’ g (95% CI)	CASPER controlHedges’ g (95% CI)
PHQ-9	0.86 (0.46 to 1.30)	0.54 (0.41 to 0.67)	0.21 (0.10 to 0.34)
GAD-7	1.0 (0.52 to 1.50)	0.33 (0.22 to 0.44)	0.11 (0.01 to 0.21)
SF-12 (physical component)	0.40 (0.03 to 0.79)	−0.02 (−0.14 to 0.09)	0.05 (−0.06 to 0.16)
SF-12 (mental component)	−1.13 (−1.70 to −0.62)	−0.28 (−0.40 to −0.16)	−0.05 (−0.16 to 0.06)

GAD-7, Generalized Anxiety Disorder-7; PHQ-9, Patient Health Questionnaire-9; SF-12, 12-item Short-Form Health Survey (version 2).

### Qualitative findings

Acceptability of, and satisfaction with, the intervention was investigated with the application of the TFA^21^ as a thematic analytical lens. The TFA defines acceptability as ‘a multi-faceted construct that reflects the extent to which people delivering or receiving a healthcare intervention consider it to be appropriate, based on anticipated or experienced cognitive and emotional responses to the intervention’. The framework consists of seven conceptual constructs related to the acceptability of interventions (figure 1). Findings from the TFA analysis incorporating both participant and coach perspectives are presented under each construct, below.

#### Affective attitude

Affective attitude relates to how an individual feels about the intervention and was explored in terms of study participants’ and coaches’ views on receiving or delivering BALM, reasons for participating, and how individuals ‘felt’ about their overall experience. Evident in the data were a range of internal and external factors associated with participants’ and coaches’ attraction to the programme. For male study participants, prominent themes included recognising a need to do something about their mood, the appeal of a mental health programme with a specific focus on men, confidentiality, and the nature of the intervention on developing the ‘tools’ to help tackle work-related stress. Several study participants also described the appeal of the practical, action-oriented nature of BALM compared with more traditional talking-based therapies.

It wasn’t like counselling, it didn’t look like a sit and talk to a counsellor and someone will fix all your problems for you. It was more the fact that it was a tool that…even when the programme was finished, it was something that if it worked for me, I had nothing to lose, I might as well give it a go and see if it’s something that will help me and work. (Y0001)

BALM coaches predominantly described a positive affective attitude in relation to a desire to help others working in the NHS as well as the opportunity to develop their own knowledge, skills and experience.

Ultimately what drives me is wanting to create a better quality of life for people, so addressing where there’s need and addressing where there’s areas where people’s quality of life isn’t so great. (CT24)

#### Intervention coherence

Intervention coherence was the extent to which study participants and coaches understood the BALM programme and how it worked. The overall aim of BALM and the principles of BA appeared to be well understood by participants. Key themes included the simplicity of the principles underpinning BA and the relationship between behaviour and emotional state, which resonated widely with men.

I found it really simple, really, really simple to follow. I think what I will take from the sessions that we did was that there was actually no need to go on and complicate matters…I think we just actually need to give the simple plans a good try, give it the opportunity to work. (T0028)

All coaches commented positively on their training and how it had helped to develop a clear understanding of how BA works. Confidence and coherence of the intervention were enhanced by the provision of ongoing support from the research team and clinical supervision.

I felt confident, obviously, you feel a bit nervous, don’t you? But I felt confident that, do you know what, I can do this. I’ve got my resources. I’ve been on the training. I’ve got help. I’ve got the help [telephone] numbers if anybody is at risk on the appointment. (CY07)

#### Burden

The burden construct addressed the perceived amount of effort required to participate in BALM. The majority of male study participants who completed the full BALM programme did not perceive the telephone sessions with coaches to be a burden, with flexibility and the ability to schedule and/or reschedule sessions at convenient times a prominent theme in men’s experiences.

Really reasonable, actually, it [commitment to do intervention] wasn’t too much. And I think there was quite a lot of flexibility with the coach as well, so between us, we sort of discussed it. Do you want it to be weekly or the same day every week or…? So it was flexible, it was good…I can’t say there was [any burden], to be honest. (Y0001)

Undertaking ‘homework’ between telephone sessions, for example reintroducing meaningful activities such as physical exercise or seeing friends and family, was generally described by men as the main ‘effort’ required to participate in BALM, though this was not seen as burdensome. However, the interviews with participants who did not complete the full programme reported that a lack of time and ability to commit to fitting in sessions with their coach over the duration of the programme was a common reason cited for withdrawing from the BALM sessions. These insights correspond to the secondary outcome of adherence and barriers to completion identified in the trial registry:

…so it’s just, it was really having to arrange the [coach] sessions and to do them and not having the time to do them that was the real issue… and I realised it just wasn’t going to work, so, you know, it wasn’t going to happen. (T0019)

Most coaches reported that they were unable to make adequate adjustments to their usual working role to accommodate their BALM coaching commitments. As such, finding sufficient time to prepare for sessions, maintain contemporaneous records and schedule phone calls with participants was commonly highlighted by coaches to be burdensome, with many finding it impinged on their free time outside of work.

Sometimes I found it quite, just intrusive into my own free time. And sometimes it stopped me doing other things that I actually needed to do. I was having to, oh no, right, I’ve got to be around and I need to make sure I do this. And then when [a session] didn’t happen because I couldn’t get hold of people it’s oh God, right, well I’ll have to reorganise that for next week or I’ll have to try and reorganise that for tomorrow. (CY20)

#### Opportunity costs

Opportunity costs are defined as the extent to which benefits, profits or values had to be given up to engage with the intervention. In terms of intervention delivery, there were mixed views in how study participants preferred to schedule phone calls with coaches to minimise opportunity costs. Some men accommodated calls during their working day, while others preferred to engage with BALM coaching sessions during their personal time. Several men described having no choice but to take calls outside of work because of the demands of their job role. One participant discussed the difficulty in finding a mutually convenient time for sessions with his coach due to his inability to take calls at work, reflecting a similar theme prominent in the analysis of data from coach interviews. Conducting sessions over the telephone was widely seen as beneficial by participants as it provided flexibility and convenience, but also confidentiality and a ‘safe space’ to talk that afforded a level of anonymity (see also Ethicality, below).

For me, [coaching sessions] over the telephone is a little bit easier…It’s a little bit, I don’t know how to describe it. A little bit kind of less personal, or you feel that you can, you know, be a little bit more maybe open or honest. With the phone as well, you don’t need to have internet connectivity. So, you’re not tied to just being in the house where you’ve got wi-fi or whatever it is…over the phone, you could be anywhere. You can go and drive off in your car and have 30 minutes…Because that’s what I did last week when I had it. I said to the Mrs, I said, right, I said, I’m going out. The kids are home. Are you all right looking after the kids just for, while I nip to B&Q? So, I went all the way to B&Q and just pulled over. [My coach] phoned us up, we had a chat, 30 odd minutes, and then I carried on to B&Q. (N0023)

#### Self-efficacy

Self-efficacy was defined as how confident male study participants and their coaches felt in undertaking the behaviours associated with the BALM process. Participants often discussed self-efficacy in relation to key BA principles such as increasing access to healthy behaviours, reducing avoidance behaviours, and understanding and attempting to address barriers to activation. Although not universal, awareness and understanding of the link between changing patterns of behaviour and improving mood were commonly highlighted in men’s accounts. For example, participants discussed being able to better recognise signs that they may be struggling with their mental health and behaviours that did not help their mood in the long term.

I think I got to the stage where I was locking myself away. So often, when I was on night shifts and that as well, I’d sleep in a separate room from my wife…. And I find myself, if I’m waking up, even at a decent time, I’ll just be like, no, I’m staying in here. The kids are downstairs, the wife is downstairs and it’s a weekend and stuff, I don’t want to get involved in family activities because I’m just thinking, this is my little protective bubble. And I think that’s one of the things that I need to get out of, and that’s one of the things that I’m trying to do. I’m trying to kind of have more kind of family time really and trying to turn the family time into positive experiences rather than just the roller coaster of just levels of frustration. (N0023)

Several coaches described how their confidence in delivering the intervention had increased as they became more experienced. Some also discussed adopting aspects of the intervention to benefit their own mental health. Although low self-efficacy was not a prominent feature of the data, there were some examples where coaches encountered difficulties in taking a collaborative approach to developing a treatment plan with participants.

Not that I was wanting to tell them what to do, but I was having to stop myself proposing different types of ideas, “have you thought about it…”, rather than getting them to do things. (CY20)

#### Ethicality

The ethicality construct related to what participants valued most or least about the BALM intervention and its ‘fit’ with their needs. For men who received the intervention, having the opportunity to address their mental health by focussing on behaviour change as opposed to ‘just talking’ was the most prominent feature of ethicality. For some men, the action-oriented nature of BALM also helped overcome the perceived stigma of engaging with mental health support. BALM being male-specific, confidential, and provided over the phone were also highly valued aspects of the intervention.

I think the fact that it was over the phone, sort of, I don’t know, it helps in a way because it keeps that, sort of, distance. Well it did for me anyway, I just feel if I’d seen the, you know, seen the person then there’s the potential to then bump into him and just feel, or whatever. (Y0009)

Frequently reported by coaches was a lack of mental health and well-being support for NHS staff in general, which BALM was perceived as helping to fill that gap. Negative views of the pressures of working in the NHS were often shared.

The NHS used to be a job to the grave. Now it’s putting people in graves. In my opinion. (CN01)

#### Perceived effectiveness

To determine perceived effectiveness, the extent to which BALM was perceived as likely to achieve its purpose, participants and coaches were asked about their views on the usefulness of the intervention, its potential viability in the wider NHS, and any recommendations for improvements. Both participants and coaches shared the view of the importance of providing BALM at scale in the NHS.

I’d be lying if I said it’s been a miracle cure…But it’s given me an outlet and it’s given me a mechanism of being able to speak to somebody who kind of gets me. Who is not, sorry, who is totally impartial. It’s not the wife who has got her own agenda with things and not the kids whose kind of wanting things…So, having somebody that’s impartial that I can speak to, that kind of gets it, has been really beneficial to me firstly. I think that’s helped me to change things, and recognise and think, you know. (N0030)

Suggestions for improvement mostly centred on the development of technology associated with the intervention materials. Most relevant here were the diary and goal-setting activities in the BALM booklet/manual, with several participants suggesting accessibility could be improved with the development of an app to replace the need to write on paper. This was viewed by one participant as something that would also help with confidentiality.

I’m not great at picking up literature and, you know, using kind of booklets or keeping a diary and stuff. That might work for a lot of other different people. And so, if there’s any kind of different ways in which delivery of progress can be kind of monitored…Even if there’s like an app that could be developed where you can just, oh, today I’ll put in this activity. Rather than keeping a physical kind of paper file. Because, as much as I love my wife, I wouldn’t have wanted her nosing over my stuff, if you know what I mean. (N0023)

## Discussion

In this study, we evaluated the impact of a tailored, gender-responsive mental health intervention for improving depression and anxiety in male frontline NHS workers. We also sought participants’ and coaches’ views about the acceptability of the intervention. We found that the BALM intervention was acceptable for men with less severe depression and anxiety working in frontline NHS roles and contributed to improvements in depression, anxiety and quality of life (mental component), which were sustained at 6-month follow-up. The quality of life (physical component) score showed a temporary worsening of physical health at 4 months, though this had returned to baseline levels at 6 months. The benchmarking data indicated that the outcomes at 4 months compared well with another active intervention and clearly exceeded the level of improvement in the control condition of the randomised trial.

The study has several strengths. It is the first to evaluate the impact and acceptability of a BA intervention for improving depression and anxiety in men using a gendered lens, contributing novel findings of relevance beyond the study context. The use of the TFA to inform data collection and analysis enabled a theoretically informed, systematic assessment of the acceptability of the intervention. Rich data were gathered from qualitative interviews with a substantial sample of male participants and coaches who supported intervention delivery, including participant non-completers.

The study also has important limitations. Participants were a self-selecting sample of men who may have been particularly motivated to take part in the intervention. It is possible that the views expressed by study participants were more positive than those of the wider population of male NHS staff. Our sample also lacked ethnocultural diversity and representation of some NHS staff groups, particularly ancillary roles, which limits the transferability of the findings. In addition, while coaches did not conduct interviews with their own participants, the fact that interviews were conducted by members of the wider research team who were also involved in overseeing delivery of the intervention may have influenced participant responses. Further, we did not assess potential differences in outcome based on the mode of intervention delivery (telephone vs video), which may have affected participant experience and engagement.

The small sample size and use of a single-arm, pre-post design mean the study was not powered to detect statistically significant changes and is not suitable for drawing causal inferences. Quantitative outcomes were analysed using a completer analysis rather than intention-to-treat, which may also introduce bias. However, BA is a well-established intervention for depression, and the pre-post results we achieved in the study are comparable to the pre-post effects seen in the intervention arm, and greater than those seen in the control arm, of high-quality trials of BA delivered using a similar format.^15^ These findings are also consistent with the results from two meta-analyses on the effectiveness of BA, including in low-intensity, non-specialist formats similar to BALM.^14 22^ The effect sizes observed in our study are within the range reported in these reviews, supporting the intervention’s potential as a brief, scalable approach for addressing depression and anxiety in men in occupational settings.

It is unclear how to interpret the temporary worsening of the physical component of the quality of life measure at 4 months. One possibility is that this is just a chance finding. Another is that as people start to make changes and increase physical activity, they temporarily feel physically worse. This, though, has not been observed in other studies of BA. The impact on physical well-being should be examined in future studies of BA.

Overall, our study findings highlight three key points of discussion. First, with levels of depression and anxiety rising in the NHS, and men’s lower participation in existing mental health programmes, our study shows the potential of BALM to help address the urgent need for early intervention and preventative mental health services outlined in the NHS Long Term Workforce plan.^27^ Most interventions implemented to date have had a focus on individual resilience with content including creative practices, emotion regulation activities, psychoeducation and mindfulness.^3 5^ However, these are likely to have limited efficacy as the overall resilience level of healthcare workers is already high.^28^ Our study has demonstrated that an evidence-based treatment programme for depression and anxiety can be tailored to be acceptable to, and beneficial for, male NHS frontline workers. Although interventions designed to alter individual health behaviours have received criticism as a reactive strategy to occupational stress that is more appropriately addressed through organisational change,^1 3^ there is good evidence that common mental health problems are preventable and treatable in the workplace with appropriate individual intervention. Workers who receive treatment for stress, depression and anxiety through workplace well-being interventions report higher levels of job satisfaction,^29^ and the NHS Long Term Workforce plan notes that investment of £80 per member of staff in mental health support can achieve net gains of £855 a year through savings from absenteeism and presenteeism.^27^

Second, our study is the first to show that tailoring a BA intervention using a gendered lens^19^ can yield benefit in men’s engagement with mental health support. BALM was perceived as acceptable by male participants across all constructs of the TFA. Tailoring interventions to make them more aligned with men’s needs and preferences has been widely suggested as a solution to men’s more reluctant attitude towards engaging with mental health services.^30^ Despite considerable research in this area, there remains little evidence on effective interventions that address these challenges. Our study has demonstrated that the practical and action-oriented strategies of BALM were highly valued by and appealing to men, highlighting its promise as a mental health intervention for the broader male population. Our findings resonate with previous suggestions in the literature that collaborative approaches that are solution-focused, and allow men to enact an aptitude for problem-solving and self-management over passive dependency, can improve engagement.^11^ Other reported reasons for taking part in BALM were also consistent with factors reported in the wider literature on improving men’s engagement with psychological support. These included the appeal of a mental health programme focused on and marketed specifically for men, assurances on confidentiality and anonymity, and flexibility and convenience. Of particular relevance is the use of the telephone for intervention delivery. Coach sessions delivered over the telephone were considered convenient and highly acceptable by both male participants and coaches, replicating findings from previous studies of BA delivered in a similar format.^31^ In addition to offering further support to evidence that telephone-delivered mental health support does not negatively affect participant-coach interactions,^32^ our study suggests that it may actually enhance accessibility and the quality of therapeutic relationships for some male service users. However, our results do suggest that some minor modifications to our clinical protocol may further enhance acceptability, in particular providing protected time to enable staff to accommodate their BALM coaching commitments, and the provision of tech-based (app) functionality for interactive elements of the intervention such as diary keeping, mood tracking and activity scheduling.

Finally, on the basis of this initial study, BALM could have particular value as an occupational well-being provision outside the NHS, specifically in other frontline and emergency response communities such as police, military and fire services, and in male-dominated occupations. Although there is high prevalence and diversity of men’s mental health challenges across a broad spectrum of workplaces and industries, the prevalence of depression within frontline and male-dominated workforces (>70% male employees) is substantially higher than in the general workforce.^9^ In addition to mental health risks arising from occupational characteristics of male-dominated and frontline roles, these work environments can foster ‘masculinity contest cultures’ that enhance social pressures to conform to gender norms that are associated with reticence to seek help, lower mental health literacy and higher mental health-related stigma.^33 34^ Evidence on effective early or preventative mental health interventions in these workplaces is limited, particularly those which incorporate gender transformative approaches that ‘work with and rework’ traditional masculine norms.^19 35^ The current study offers a platform for future research in these higher risk settings.

## Conclusion

This pre-post intervention study has shown that the delivery of a tailored, gender-responsive BA intervention was appealing to, and beneficial for, men working in frontline NHS roles with less severe depression and anxiety. The practical and action-oriented strategies of the intervention, and the confidential, flexible, convenient mode of delivery, worked to support men’s engagement with the intervention. The BALM intervention offers promise as a tailored workplace mental health programme that is aligned with men’s needs and preferences and can help overcome a reticence to engage with mental health support in NHS staff and beyond.

## Data Availability

Data are available upon reasonable request. All data relevant to the study are included in the article or uploaded as supplementary information.
