# Supplementary material for: Behavioural activation for low mood and anxiety in male frontline NHS workers (BALM): a pre-post intervention study
Source: BMJ Open. 2025 Jun 12;15(6):e094214. doi: 10.1136/bmjopen-2024-094214 (PMC12182024; doi:10.1136/bmjopen-2024-094214)
Supplement: Supplementary file 1 [file bmjopen-2024-094214.reviewer_comments.pdf]

PEER REVIEW HISTORY

BMJ Open publishes all reviews undertaken for accepted manuscripts. Reviewers are asked to complete a checklist review form and are provided with free text boxes to elaborate on their assessment. These free text comments are reproduced below.

ARTICLE DETAILS

Title (Provisional)

Behavioural activation for low mood and anxiety in male frontline NHS workers (BALM): a pre-post intervention study

Authors

Galdas, Paul; Bailey, Della; Bell, Steve; Bosanquet, Katharine; Chew-Graham, Carolyn; Ekers, David; Gilbody, Simon; Littlewood, Elizabeth; Mawhinney, Michael; Stevens, Heidi; Webb, Katie; McMillan, Dean

---

VERSION 1 - REVIEW

**Reviewer**	**1**
**Name**	**Schmitt , Julia C.**
**Affiliation**	**Universidad Nacional de Educación a Distancia, Departamento de Personalidad, Evaluación y Tratamientos Psicológicos**
**Date**	**16-Dec-2024**
**COI**	**None**

It has been a pleasure to review your manuscript which I consider of interest to the research community and of high practical relevance. Below, I will outline some minor issues that should be resolved before publishing the paper.

- “3. Health-related quality of life measured using the SF-12 questionnaire” => mentioned at trial registration, but not in the article. Why? This should be explained.

- Introduction
  - ⇨ “As BALM was an early mental health intervention, individuals were eligible for participation in this study if they scored in the subclinical range (5-14) on the Patient Health Questionnaire-9 (PHQ-9) and/or the Generalised Anxiety Disorder-7 questionnaire (GAD-7) at baseline.” => Include reference.
  - ⇨ The different measurement time points should be justified (at least the follow-up time points, e.g. including a reference to similar studies; or acknowledging the lack of longer follow-up periods etc.): pre-treatment, four months, and six months.

- When the intervention “BALM” is described, present tense should be used.

- Intervention
  - ⇨ Video or telephone calls according to preference = this might have differential effects. Have those been looked at?
  - ⇨ “up to” eight phone calls => wasn’t that standardized? Indicate mean number and range of phone calls; duration; 4-6 weeks => seems to be a range, different dose of intervention?
  - ⇨ As BALM is a new intervention, more information about its content should be given: content and structure of the 8 sessions, content and use of the self-help manual.

- Outcomes
  - ⇨ The three instruments used in the study should be described in more detail.
  - ⇨ “Gilbody S, Lewis H, Adamson J, et al. Effect of Collaborative Care vs Usual Care on Depressive Symptoms in Older Adults With Subthreshold Depression: The CASPER Randomized Clinical Trial. JAMA : the journal of the American Medical Association 2017;317(7):728-37. doi: 10.1001/jama.2017.0130” => Maybe find another, more appropriate (as in similar) study to compare with?

- Sample size
  - ⇨ Reasons for achieving 45 participants do not seem convincing. If only 45 participants could be achieved through recruitment, this should just be stated, not described as a previous aim. Otherwise, previous sample size calculations should have been done.
- “4- and 6-months.” => “after four and six months”

- Data collection
  - ⇨ Was it the same coach who conducted the intervention and the interview? If it was, then it should be stated as a limitation of the study.

- Analysis
  - ⇨ A statistical procedure for repeated measures should be used, given the small sample size non-parametric (if the data does not follow a normal distribution), such as the Wilcoxon signed rank test.
  - ⇨ “For an effect size to be calculated, data was **need** for each time point on the measure.” => Correct the sentence grammatically.

- Figure 1
  - ⇨ Maybe provide all the information of the different constructs in this figure? Or design as a table?

- “Recruitment and participant flow” should rather go into the “Data collection” section than stay in the “Results” section

- Results
  - ⇨ Recruitment and participant flow
    - o Were reasons for non-completion/abandonment registered?
    - o Justify, why 4 coach sessions were established to reach completer status.

- The participant flow diagram should include the follow-up time points and reasons for abandonment or exclusion at the different time points.

⇨ What kind of analysis were conducted: intention-to-treat or completer analysis?

⇨ The section "Baseline data" should rather be called "Demographic characteristics" as this is what is described in this paragraph.

⇨ Quantitative outcomes
  - Check the N in table 1 as there seem to be more participants at the six-month follow-up than earlier in the study, which does not make sense.
  - "Mean (SD) should go into the columns (not in the rows)

⇨ Qualitative findings
  - Takes up much space of the article (compared to the rest) and might be shortened.
  - Why were not all the participants interviewed about their satisfaction with the program?
  - "In total, 32 study participants were selected via consecutive sampling and interviewed from the pool of 45 study participants who consented to take part in an interview at initial recruitment: 24 were 'completers' and 8 were 'non-completers'. Twelve coaches were also selected for interview." => This should go into the Data collection section
  - "Acceptability of, and satisfaction with the intervention was investigated with the application of the TFA19 as a thematic analytical lens. The TFA defines acceptability as 'a multi-faceted construct that reflects the extent to which people delivering or receiving a healthcare intervention consider it to be appropriate, based on anticipated or experienced cognitive and emotional responses to the intervention'. The framework consists of seven conceptual constructs related to the acceptability of interventions (figure 1)." => This should go into the Outcomes section.
  - "Affective attitude relates to how an individual feels about the intervention and was explored in terms of study participants' and coaches' views on receiving or delivering BALM, reasons for participating, and how individuals 'felt' about their overall experience." => This should go into the Outcomes section.
  - "Intervention coherence was the extent to which study participants and coaches understood the BALM programme and how it worked." => This should go into the Outcomes section.
  - "The burden construct addressed the perceived amount of effort required to participate in BALM." => This should go into the Outcomes section.
  - "Opportunity costs are defined as the extent to which benefits, profits or values had to be given up to engage with the intervention." => This should go into the Outcomes section.
  - "Self-efficacy was defined as how confident male study participants and their coaches felt in undertaking the behaviours associated with the BALM process." => This should go into the Outcomes section.
  - "The ethicality construct related to what participants valued most or least about the BALM intervention and its 'fit' with their needs." => This should go into the Outcomes section.
  - "To determine perceived effectiveness, the extent to which BALM was perceived as likely to achieve its purpose, participants and coaches were asked about their views on the usefulness of the intervention, its potential viability in the wider NHS, and any recommendations for improvements."

=> This should go into the Outcomes section.

- Discussion
  - ⇨ The paragraph about the strengths and limitations of the study should go to the end of the Discussion section.
  - ⇨ The results should be compared more with other, similar intervention studies.
  - ⇨ “Rich data were gathered from qualitative interviews with a large sample of male participants and coaches who supported intervention delivery, including participant non-completers.” => It certainly is not a large sample.
  - ⇨ “It is possible their views on the acceptability of BALM are more positive than the wider population of male NHS staff.” => Check grammar.

- References
  - ⇨ “Ekers D, Richards D, Gilbody S. A meta-analysis of randomized trials of behavioural treatment of depression. Psychological medicine 2007;38(5):611-23. doi: 10.1017/S0033291707001614” => Include more recent reviews/meta-analysis/literature on this topic.

VERSION 1 - AUTHOR RESPONSE

#	Comment	Response	Manuscript Location
4	SF-12 mentioned at trial registration but not in article. Why?	The manuscript has been updated to include the SF-12 as one of the outcome measures. The SF-12 was administered at all time points, and both physical and mental component scores are now reported in the Results section, with pre-post effect sizes included in Tables 2 and 3. Interpretations of these outcomes have also been added to the Discussion.	pp.5–8; Methods: Outcomes; Results: Quantitative outcomes; Discussion
5	Include references for PHQ-9 and GAD-7.	References added	p.5; Methods: Participants & sample, second para
6	Justify the follow-up time points (4 and 6 months).	Added brief justification of the four and six month time points, including reference to comparable studies, and noted the absence of longer-term follow-up.	p.3; Methods: Study design

7	Use present tense when describing the BALM intervention.	Revised throughout to consistently use present tense.	See tracked changes throughout
8	Preference for video/telephone calls may affect outcomes - has this been analysed?	Clarified in the Intervention section that delivery mode was not standardised and outcomes by mode were not analysed. This has also been acknowledged as a limitation in the Discussion.	p.5; Methods: Intervention and p.12 Discussion
9	Clarify if number of sessions was standardised. Report mean and range.	Revised text to clarify that the number and timing of sessions were not standardised and were tailored based on participant need and preference. Duration of individual sessions is noted. Descriptive statistics on session numbers were not available.	p.5; Methods: Intervention.
10	Provide more detail on BALM intervention content and manual.	A narrative description and summary table of the BALM intervention stages and content have been added to the Intervention section to clarify the structure and key components of the self-help manual and coaching sessions.	pp.4-5 Methods: Intervention section (with new table 1)
11	Describe the three outcome instruments in more detail.	Expanded the Outcomes section to include detailed descriptions of the PHQ-9, GAD-7, and SF-12.	p.5; Methods: Outcomes
12	Consider if there is a more appropriate comparator than CASPER trial.	We recognise the CASPER trial differs in population and setting. However, we have retained it as the most appropriate comparator available due to its use of a low-intensity Behavioural Activation intervention in the form of a brief, guided self-help programme, and because the sample had subclinical levels of symptomatology. Few trials exist that closely mirror our study's population and delivery context; CASPER remains one of the most methodologically aligned options.	No amendment made.

13	Sample size justification not convincing.	This was a single-arm, pre-post intervention study and not an RCT. As such, no formal power calculation was required. The target sample size of 45 participants was pragmatically set to enable benchmarking against similar BA interventions and to ensure sufficient qualitative data to achieve meaning saturation. We have retained this framing as it reflects the methodological approach appropriate for this study design.	No amendment made.
14	Use "after four and six months" instead of "4- and 6-months".	Revised.	See tracked changes throughout.
15	Clarify if same coach conducted intervention and interviews. If so, note as limitation.	Clarified that all interviews were conducted by members of the research team who were not the participants' coaches.	p.5; Methods: Data Collection
16	A statistical procedure for repeated measures should be used, given the small sample size non-parametric (if the data does not follow a normal distribution), such as the Wilcoxon signed rank test	As this was a quasi-experimental study, the analysis focused on estimating pre-post effect sizes (Hedges' g) rather than testing statistical significance. No inferential tests (e.g. Wilcoxon) were conducted, in line with recommendations for exploratory intervention studies.	No amendment made.
17	Correct grammar: "data was need"	Revised to "data were needed".	p.6; Methods: Quantitative analysis
18	Figure 1 - consider table instead.	Revised figure to improve clarity.	Figure 1
19	Move "Recruitment and participant flow" from Results to Data Collection section.	We have retained this content in the Results section in line with CONSORT guidance, which recommends presenting participant flow and recruitment outcomes as part of study results.	No amendment made.
20	Report reasons for non-completion.	Reasons for non-completion were not formally registered. However,	No amendment made.

		qualitative interviews were conducted with participants who did not complete the intervention, and these data provided insight into common barriers to completion.	
21	Justify why 4 coach sessions was threshold for “completer”.	Added a clarification that completer status was defined as attending at least four coach sessions, which was typically the minimum required to progress through the five structured stages of the BALM intervention.	p.6; Results: Recruitment and Participant flow
22	Participant flow diagram should include follow-ups and reasons for dropout.	As this was a single-arm pre-post intervention study, reasons for loss to follow-up were not systematically recorded and are not included in the flow diagram. Completion rates for follow-up assessments at each time point are reported in Table 2. Reasons for non-completion were explored qualitatively with participants who did not complete the intervention.	No amendment made.
23	Specify whether analysis was intention-to-treat or completer analysis.	Added a sentence clarifying we employed a completer analysis approach consistent with the study's exploratory objectives.	p.6; Methods: Quantitative analysis
24	Rename “Baseline data” section to “Demographic characteristics”.	We have retained the heading “Baseline data” in line with CONSORT reporting guidance which recommends this label for sections presenting demographic and clinical characteristics.	No amendment made.
25	Table 1 has more participants at 6 months than earlier - explain.	Added a sentence clarifying that the higher N at 6 months reflects the flexible and rolling nature of data collection, with some participants completing the 6-month follow-up despite missing the 4-month assessment.	p.7; Results: Quantitative Outcomes
26	Move “Mean (SD)” to columns, not rows.	Table 1 has been reformatted to display Mean (SD) values within	p.7; Table 2

		the column headers, as recommended.	
27	Consider shortening qualitative findings section.	The Qualitative Findings section has been streamlined by reducing the number of illustrative quotes to improve overall manuscript balance while retaining the richness and integrity of the TFA-based analysis.	pp.8-12; Results: Qualitative findings
28	Why weren't all participants interviewed?	In line with conventions for qualitative research, the aim was not to interview all participants but to gather rich, diverse data sufficient to explore the acceptability of the intervention. Consecutive sampling was used among those who consented to interview, and interviews continued until meaning saturation was judged to have been achieved.	No amendment made
29	Move participant/coach sampling details to Data Collection section.	Sentence describing the qualitative sample and selection process has been moved from the Results to the Data Collection section.	pp.5-6; Methods: Data Collection
30	TFA construct definitions and applications should go in Outcomes, not Results.	We have retained the TFA definitions and construct explanations within the Qualitative Findings section, as they were applied thematically to structure the analysis. The framework was used as an interpretive lens during data analysis rather than as a predefined outcome measure.	No amendment made
31	Strengths and limitations paragraph should be at the end of the Discussion.	We have retained the strengths and limitations paragraph in its current position in line with accepted reporting conventions for pre-post studies and the editorial style of BMJ Open. We believe that positioning this content immediately after the summary of key findings allows for	No amendment made

		clearer interpretation of results and maintains logical flow into the implications and future research directions, which are typically discussed at the end of the Discussion section.	
32	The results should be compared more with other, similar intervention studies.	Discussion has been expanded to include comparisons with two recent meta-analyses which evaluate the effectiveness of Behavioural Activation, including in low-intensity and non-specialist delivery formats similar to BALM. This provides a clearer context for interpreting the intervention's effect sizes and aligns BALM with the broader evidence base.	p.12; Discussion
33	"Large sample" of interviewees is overstated.	Revised the wording to describe the sample as 'substantial' which more accurately reflects its strength within a qualitative research context.	p.12; Discussion
34	"It is possible their views..." sentence needs grammar check.	Revised for clarity and grammar.	p.12; Discussion
35	Update 2007 meta-analysis citation with more recent literature.	We have cited more recent and comprehensive reviews: Ekers et al. (2014) and Uphoff et al. (2020). These provide updated meta-analytic evidence on the effectiveness of BA, including subgroup analyses relevant to delivery mode and intervention setting.	References
